# Gene model for the ortholog of *lin-28* in *Drosophila simulans*

**DOI:** 10.17912/micropub.biology.000963

**Published:** 2025-06-04

**Authors:** Megan E. Lawson, Hannan Saeed, Cassie Tran, Simran Chhina, Jack A. Vincent, Brian Schwartz, Kellie S. Agrimson, Christopher E. Ellison, Chinmay P. Rele, Laura K Reed

**Affiliations:** 1 University of Alabama, Tuscaloosa, Alabama, United States; 2 University of Washington Tacoma, Tacoma, Washington, United States; 3 Columbus State University, Columbus, Georgia, United States; 4 St. Catherine University, Saint Paul, Minnesota, United States; 5 Rutgers, The State University of New Jersey, New Brunswick, New Jersey, United States; 6 The University of Alabama, Tuscaloosa, AL USA

## Abstract

Gene model for the ortholog of *lin-28* ( *lin-28* ) in the May 2017 (Princeton ASM75419v2/DsimGB2) Genome Assembly (GenBank Accession: GCA_000754195.3 ) of *Drosophila simulans* . This ortholog was characterized as part of a developing dataset to study the evolution of the Insulin/insulin-like growth factor signaling pathway (IIS) across the genus *Drosophila* using the Genomics Education Partnership gene annotation protocol for Course-based Undergraduate Research Experiences.

**Figure 1. Genomic neighborhood and gene model for lin-28 in Drosophila simulans:**
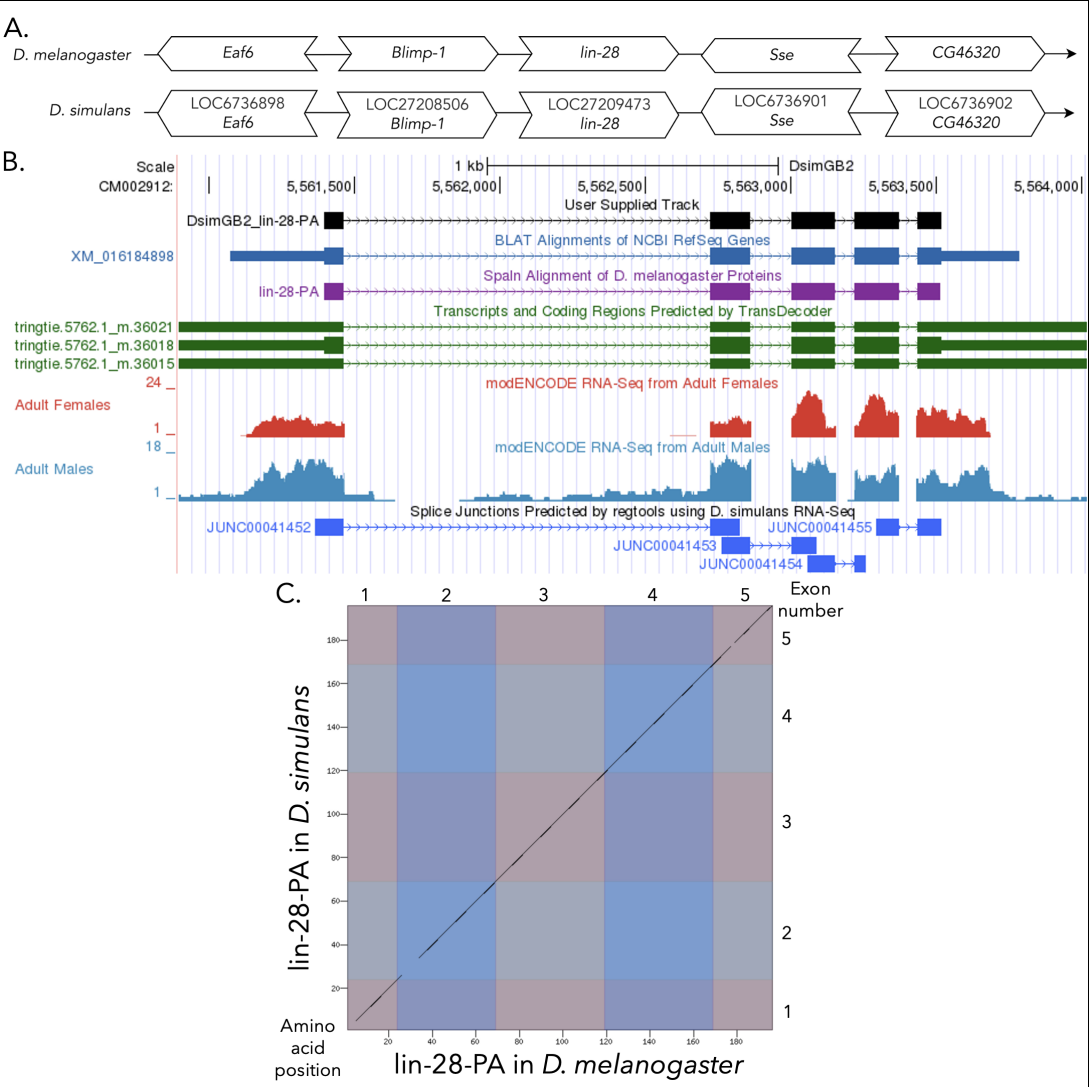
**
(A) Synteny comparison of the genomic neighborhoods for *lin-28* in *Drosophila melanogaster* and *D.* *simulans* .** Thin underlying arrows indicate the DNA strand within which the reference gene– *lin-28* –is located in *D. melanogaster* (top) and *D. simulans* (bottom). Thin arrows pointing to the right indicate that *lin-28* is on the positive (+) strand in *D. melanogaster* and *D. simulans* . The wide gene arrows pointing in the same direction as *lin-28* are on the same strand relative to the thin underlying arrows, while wide gene arrows pointing in the opposite direction of *lin-28* are on the opposite strand relative to the thin underlying arrows. White gene arrows in *D. simulans* indicate orthology to the corresponding gene in *D. melanogaster* . Gene symbols given in the *D. simulans* gene arrows indicate the orthologous gene in *D. melanogaster* , while the locus identifiers are specific to *D. simulans* . **(B) Gene Model in GEP UCSC Track Data Hub** (Raney et al., 2014). The coding-regions of *lin-28* in *D. simulans* are displayed in the User Supplied Track (black); coding CDSs are depicted by thick rectangles and introns by thin lines with arrows indicating the direction of transcription. Subsequent evidence tracks include BLAT Alignments of NCBI RefSeq Genes (dark blue, alignment of Ref-Seq genes for *D. simulans* ), Spaln of *D. melanogaster* Proteins (purple, alignment of Ref-Seq proteins from *D. melanogaster* ), Transcripts and Coding Regions Predicted by TransDecoder (dark green), RNA-Seq from Adult Females and Adult Males (red and light blue, respectively; alignment of Illumina RNA-Seq reads from *D. simulans* ), and Splice Junctions Predicted by regtools using *D. simulans* RNA-Seq (SRP006203). Splice junctions shown have a minimum read-depth of 10 with 10-49 supporting reads shown in blue. **(C) Dot Plot of lin-28-PA in *D. melanogaster* ( *x* -axis) vs. the orthologous peptide in *D. simulans* ( *y* -axis).** Amino acid number is indicated along the left and bottom; CDS number is indicated along the top and right, and CDSs are also highlighted with alternating colors.

## Description

**Table:** 

* This article reports a predicted gene model generated by undergraduate work using a structured gene model annotation protocol defined by the Genomics Education Partnership (GEP; thegep.org ) for Course-based Undergraduate Research Experience (CURE). The following information in this box may be repeated in other articles submitted by participants using the same GEP CURE protocol for annotating Drosophila species orthologs of Drosophila melanogaster genes in the insulin signaling pathway. * "In this GEP CURE protocol students use web-based tools to manually annotate genes in non-model *Drosophila* species based on orthology to genes in the well-annotated model organism fruitfly *Drosophila melanogaster* . The GEP uses web-based tools to allow undergraduates to participate in course-based research by generating manual annotations of genes in non-model species (Rele et al., 2023). Computational-based gene predictions in any organism are often improved by careful manual annotation and curation, allowing for more accurate analyses of gene and genome evolution (Mudge and Harrow 2016; Tello-Ruiz et al., 2019). These models of orthologous genes across species, such as the one presented here, then provide a reliable basis for further evolutionary genomic analyses when made available to the scientific community.” (Myers et al., 2024). “The particular gene ortholog described here was characterized as part of a developing dataset to study the evolution of the Insulin/insulin-like growth factor signaling pathway (IIS) across the genus *Drosophila* . The Insulin/insulin-like growth factor signaling pathway (IIS) is a highly conserved signaling pathway in animals and is central to mediating organismal responses to nutrients (Hietakangas and Cohen 2009; Grewal 2009).” (Myers et al., 2024). “ *D. simulans* (NCBI:txid7240) is part of the *melanogaster* species group within the subgenus *Sophophora* of the genus *Drosophila* (Sturtevant 1939; Bock and Wheeler 1972) *.* It was first described by Sturtevant (1919). *D. simulans* is a sibling species to *D. melanogaster* , thus extensively studied in the context of speciation genetics and evolutionary ecology (Powell 1990). Historically, *D. simulans* was a tropical species native to sub-Saharan Africa (Lemeunier et al., 1986) where figs served as a primary host (Lachaise and Tsacas 1983). However, *D. simulans's* range has expanded worldwide within the last century as a human commensal using a broad range of rotting fruits as breeding sites (https://www.taxodros.uzh.ch, accessed 1 Feb 2023).” (Lawson et al., 2024).

We propose a gene model for the *D. simulans* ortholog of the *D. melanogaster* *lin-28* gene. The genomic region of the ortholog corresponds to the uncharacterized protein LOC27209473 (RefSeq accession XP_016030549.1 ) in the May 2017 (Princeton ASM75419v2/DsimGB2) Genome Assembly of *D. simulans* (GenBank Accession: GCA_000754195.3 ). This model is based on RNA-Seq data from *D. simulans* ( SRP006203 ; Graveley et al., 2011) and *lin-28* in *D. melanogaster* using FlyBase release FB2023_02 ( GCA_000001215.4 ; Larkin et al., 2021; Gramates et al., 2022; Jenkins et al., 2022).

*lin-28* ( *lin-28* ) is a positive regulator of the insulin signaling (Zhu et al., 2011) and JAK-STAT (Sreejith et al., 2019) pathways. *lin-28* was discovered in *Caenorhabditis elegans* by mutations that produce heterochronic shifts in cell fate specification (Ambros and Horvitz 1984). Homologs were identified later in other animals, including *Drosophila* , *Xenopus* , mouse, and human (Moss and Tang 2003). lin-28 binds to many mRNA molecules to regulate their translation or stability (Balzer and Moss 2007; Cho et al., 2012). In *Drosophila* , lin-28 binds to the *insulin-like receptor (InR)* mRNA and stimulates the symmetric division of intestinal stem cells in response to nutrients (Chen et al., 2015; Luhur and Sokol 2015). In mammals, LIN28, in combination with OCT4, SOX2, and NANOG, can reprogram differentiated somatic cells to pluripotency (Yu et al., 2007).

***Synteny***

The referece gene, *lin-28 ,* occurs on chromosome 3L in *D. melanogaster* and is flanked upstream by *Blimp-1* and *Esa1-associated factor 6* ( *Eaf6* ) and downstream by *Separase* ( *Sse* ) and *CG46320 .* The *tblastn* search of *D. melanogaster* lin-28-PA (query) against the *D. simulans* (GenBank Accession: GCA_000754195.3 ) Genome Assembly (database) placed the putative ortholog of *lin-28* within scaffold CM002912 (CM002912.1) at locus LOC27209473 ( XP_016030549.1 )— with an E-value of 1e-42 and a percent identity of 97.67%. Furthermore, the putative ortholog is flanked upstream by LOC27208506 ( XP_016030546.1 ) and LOC6736898 ( XP_002083751.1 ), which correspond to *Blimp-1* and *Eaf6* in *D. melanogaster* (E-value: 0.0 and 2e-162; identity: 98.68% and 98.22%, respectively, as determined by *blastp* ; Figure 1A, Altschul et al., 1990). The putative ortholog of *lin-28* is flanked downstream by LOC6736901 ( XP_002083754.1 ) and LOC6736902 ( XP_016030550.1 ), which correspond to *Sse* and *CG46320* in *D. melanogaster* (E-value: 0.0 and 2e-34; identity: 95.90% and 100.00%, respectively, as determined by *blastp* ). The ortholog assignment for *lin-28* in *D. simulans* is supported by the following evidence: the synteny of the genomic neighborhood is completely conserved across both species, and all *BLAST* results used to determine orthology indicate very high-quality matches.

***Protein Model***

*lin-28* in *D. simulans* has one protein-coding isoform, lin-28-PA ( Figure 1B ). mRNA isoform *lin-28-RA* contains five CDSs. Relative to the ortholog in *D. melanogaster* , the CDS number is conserved, as *lin-28* in *D. melanogaster* also has only one RNA isoform with five CDSs. The sequence of lin-28-PA in *D. simulans* has 95.90% identity (E-value: 1e-139) with the protein-coding isoform lin-28-PA in *D. melanogaster* , as determined by *blastp* ( Figure 1C ). Coordinates of this curated gene model are stored by NCBI at GenBank/BankIt (accession **BK064527** ). These data are also archived in the CaltechDATA repository (see “Extended Data” section below).

## Methods

Detailed methods including algorithms, database versions, and citations for the complete annotation process can be found in Rele et al. (2023). Briefly, students use the GEP instance of the UCSC Genome Browser v.435 ( https://gander.wustl.edu ; Kent WJ et al., 2002; Navarro Gonzalez et al., 2021) to examine the genomic neighborhood of their reference IIS gene in the *D. melanogaster* genome assembly (Aug. 2014; BDGP Release 6 + ISO1 MT/dm6). Students then retrieve the protein sequence for the *D. melanogaster* reference gene for a given isoform and run it using *tblastn* against their target *Drosophila* species genome assembly on the NCBI BLAST server ( https://blast.ncbi.nlm.nih.gov/Blast.cgi ; Altschul et al., 1990) to identify potential orthologs. To validate the potential ortholog, students compare the local genomic neighborhood of their potential ortholog with the genomic neighborhood of their reference gene in *D. melanogaster* . This local synteny analysis includes at minimum the two upstream and downstream genes relative to their putative ortholog. They also explore other sets of genomic evidence using multiple alignment tracks in the Genome Browser, including BLAT alignments of RefSeq Genes, Spaln alignment of *D. melanogaster* proteins, multiple gene prediction tracks (e.g., GeMoMa, Geneid, Augustus), and modENCODE RNA-Seq from the target species. Detailed explanation of how these lines of genomic evidenced are leveraged by students in gene model development are described in Rele et al. (2023). Genomic structure information (e.g., CDSs, intron-exon number and boundaries, number of isoforms) for the *D. melanogaster* reference gene is retrieved through the Gene Record Finder ( https://gander.wustl.edu/~wilson/dmelgenerecord/index.html ; Rele et al *.,* 2023). Approximate splice sites within the target gene are determined using *tblastn* using the CDSs from the *D. melanogaste* r reference gene. Coordinates of CDSs are then refined by examining aligned modENCODE RNA-Seq data, and by applying paradigms of molecular biology such as identifying canonical splice site sequences and ensuring the maintenance of an open reading frame across hypothesized splice sites. Students then confirm the biological validity of their target gene model using the Gene Model Checker ( https://gander.wustl.edu/~wilson/dmelgenerecord/index.html ; Rele et al., 2023), which compares the structure and translated sequence from their hypothesized target gene model against the *D. melanogaster* reference gene model. At least two independent models for a gene are generated by students under mentorship of their faculty course instructors. Those models are then reconciled by a third independent researcher mentored by the project leaders to produce the final model. Note: comparison of 5' and 3' UTR sequence information is not included in this GEP CURE protocol.

## Data Availability

Description: GFF, FASTA, and PEP of the model. Resource Type: Model. DOI: https://doi.org/10.22002/2zvbh-fp322
